# Clues for glues: from serendipity to nature’s blueprints in degrader discovery

**DOI:** 10.1042/EBC20253058

**Published:** 2025-12-24

**Authors:** Zuzanna Kozicka

**Affiliations:** 1 Department of Medical Oncology, Dana-Farber Cancer Institute, Boston, MA, U.S.A.; 2 Broad Institute of MIT and Harvard, Cambridge, MA, U.S.A.; 3 Institute of Chemical Sciences and Engineering, École Polytechnique Fédérale de Lausanne, Lausanne, Switzerland

**Keywords:** drug discovery and design, medicinal chemistry, molecular glue degraders, targeted protein degradation, ubiquitin ligases

## Abstract

Molecular glue degraders (MGDs) are small molecules that promote interactions between an E3 ligase and a target protein, reconfiguring recognition to trigger proteasome-mediated degradation. Their discovery has so far been largely serendipitous – either recognized in retrospect or uncovered through ‘needle-in-a-haystack’ screening – but systematic strategies are beginning to emerge. This review frames two complementary routes for discovery. The first views MGDs as modular – typically anchored on either the ligase or the target – which allows the chemical search space to be biased toward such anchoring. Ligase-directed strategies derivatize known ligase binders, as demonstrated for cereblon (CRBN) and now beyond. Conversely, recent target-directed strategies remodel inhibitors into glues through solvent-exposed elaboration, effectively inverting the classical design paradigm. Both approaches tilt discovery toward chemotypes more likely to yield glue activity. Second, biology provides its own guideposts: certain protein pairs appear especially predisposed to stabilization. Endogenous degrons, mutational lesions, and transferable ‘glueprints’ of surface topology all point to contexts in which small molecules might act as functional surrogates – repairing hypomorphs, mimicking hypermorphs, or creating neomorphs. MGDs, therefore, exemplify how small molecules can reprogram recognition logic by transforming latent compatibilities into selective degradation. Together, these insights help rationalize past discoveries and suggest possible blueprints for more systematic ones ahead.

## Introduction

Targeted protein degradation (TPD) has emerged as a powerful therapeutic strategy [1,2]. By co-opting the cell’s endogenous quality-control machinery – most often the ubiquitin–proteasome system (UPS) – small molecules can trigger destruction of disease-relevant proteins. In the UPS, ~600 E3 ligases recognize substrates via degron motifs – sequential or structural elements that earmark a protein for ubiquitylation – and, together with E1 and E2 enzymes, assemble polyubiquitin chains that direct substrates to the proteasome [3,4]. Classical small-molecule drugs inhibit enzymatic activity, whereas degraders act at the level of protein fate: they eliminate the protein and silence its functions – a mode of action especially relevant for challenging targets lacking conventional active sites.

Two principal chemical strategies achieve this. Proteolysis-targeting chimeras (PROTACs) are bifunctional molecules that tether a ligase ligand to a target ligand via a linker, enforcing proximity [5,6]. Their modular architecture – separable ligands joined by a linker – makes them inherently designable. Molecular glue degraders (MGDs), by contrast, are monovalent compounds that promote ligase–substrate interactions by stabilizing co-operative protein–protein contacts [7–9]. Rather than relying on two independent ligands and high-affinity pockets, MGDs exploit interface complementarity, subtly reshaping surfaces to tip weak or latent contacts into a productive ternary complex. In practice, PROTACs and glues lie on a mechanistic continuum, but glues define its most minimal end: a single scaffold sufficient to reconfigure recognition [10]. While not bifunctional, many MGDs can nonetheless be understood in modular terms: anchored on one partner, with solvent-facing moieties bridging the interface.

The therapeutic potential of MGDs was first revealed when thalidomide’s clinical efficacy in multiple myeloma was traced to targeted degradation [11]. Subsequent examples – among them indisulam and CR8 – showed that the mechanism recurs across diverse chemotypes and targets. Since then, the field has accelerated dramatically, with databases now cataloging hundreds of examples [1,2,12]. Rather than anomalies, MGDs are increasingly seen as a recurring modality that can extend the reach of small molecules into previously less accessible biology.

This review traces how the field is moving from serendipity to systematic discovery, framed around two interlocking routes: strategies that begin from predisposed chemical matter, and principles rooted in biology that pinpoint protein pairs most amenable to stabilization. Together, these perspectives suggest that glues, once chance findings, may increasingly be approached through deliberate discovery and offer a roadmap for systematic MGD design.

## From serendipity to strategy

Thalidomide, first marketed in the 1950s and later withdrawn for its teratogenicity, re-emerged decades later as a therapy for multiple myeloma – approved even before its mechanism was understood [11]. Its true mode of action was uncovered post hoc, through a series of seminal studies. Cereblon (CRBN), a substrate receptor of the Cullin-Ring ligase 4 (CRL4) complex, was identified as the direct binding partner, and subsequent studies showed that thalidomide does not inhibit CRBN but instead endows it with neomorphic activity, reshaping its surface to recruit new substrates [13–16]. Structural work revealed how the glutarimide ring anchors in a conserved tri-tryptophan pocket of CRBN, while the phthalimide moiety projects outward to capture β-hairpin G-loop motifs, exemplified by zinc finger transcription factors IKZF1 and IKZF3 (Ikaros and Aiolos) [17–20]. Their degradation demonstrated that even a minimal synthetic scaffold can reconfigure ligase specificity, contrasting with earlier precedents such as rapamycin, for which large natural products were thought necessary to redirect protein interactions [21].

A later retrospective discovery identified indisulam, a preclinical anticancer compound, as another MGD – this time bridging RNA-binding motif protein 39 (RBM39) to DDB1- and Cullin-associated factor 15 (DCAF15) by stabilizing the insertion of a short α-helical degron [22–26]. This showed that thalidomide was not a mechanistic outlier but the first glimpse of a recurring phenomenon: small molecules may reprogram ligases by co-operatively stabilizing new interfaces. The therapeutic success of immunomodulatory drugs (IMiDs) underscored the transformative potential of this mechanism – not least because they realized the long-sought goal of inactivating transcription factors with small molecules – prompting the natural next question of how additional compounds with similar activity might be discovered systematically rather than serendipitously. Several routes have since been explored [7,27].

### Agnostic approaches

Phenotypic campaigns remain one entry point, yielding active molecules that sometimes reveal their glue mechanisms only in retrospect [22,28–30]. However, recognition of this mode of action has also prompted more directed searches: assays that probe explicitly for degradation activity while remaining agnostic to the ligase or target involved. For example, UPS dependence can be revealed through genetic or drug-sensitization contexts, such as comparing wildtype cells with neddylation-deficient cells or screening in the presence and absence of E1 or neddylation inhibitors [31,32]. A complementary approach is bioinformatic mining, in which compound sensitivity patterns across cell-line panels are correlated with ligase expression or dependency datasets to nominate candidate MGDs [33].

Despite their value, these approaches share limitations. Reported hit rates remain low (on the order of 0.1% in published screens) [31,33]; most assays use viability readouts that bias discovery toward essential targets; and mechanistic deconvolution often requires extensive proteomics, CRISPR screening, and structural follow-up. Library design further constrains outcomes: most scaffolds are optimized for pocket inhibition, while solvent-facing positions – critical for interface stabilization – are rarely diversified beyond pharmacokinetic or solubility tweaks. Taken together, these factors make agnostic searches a true ‘needle-in-a-haystack’ exercise: informative when successful but difficult to scale.

**Figure 1:**
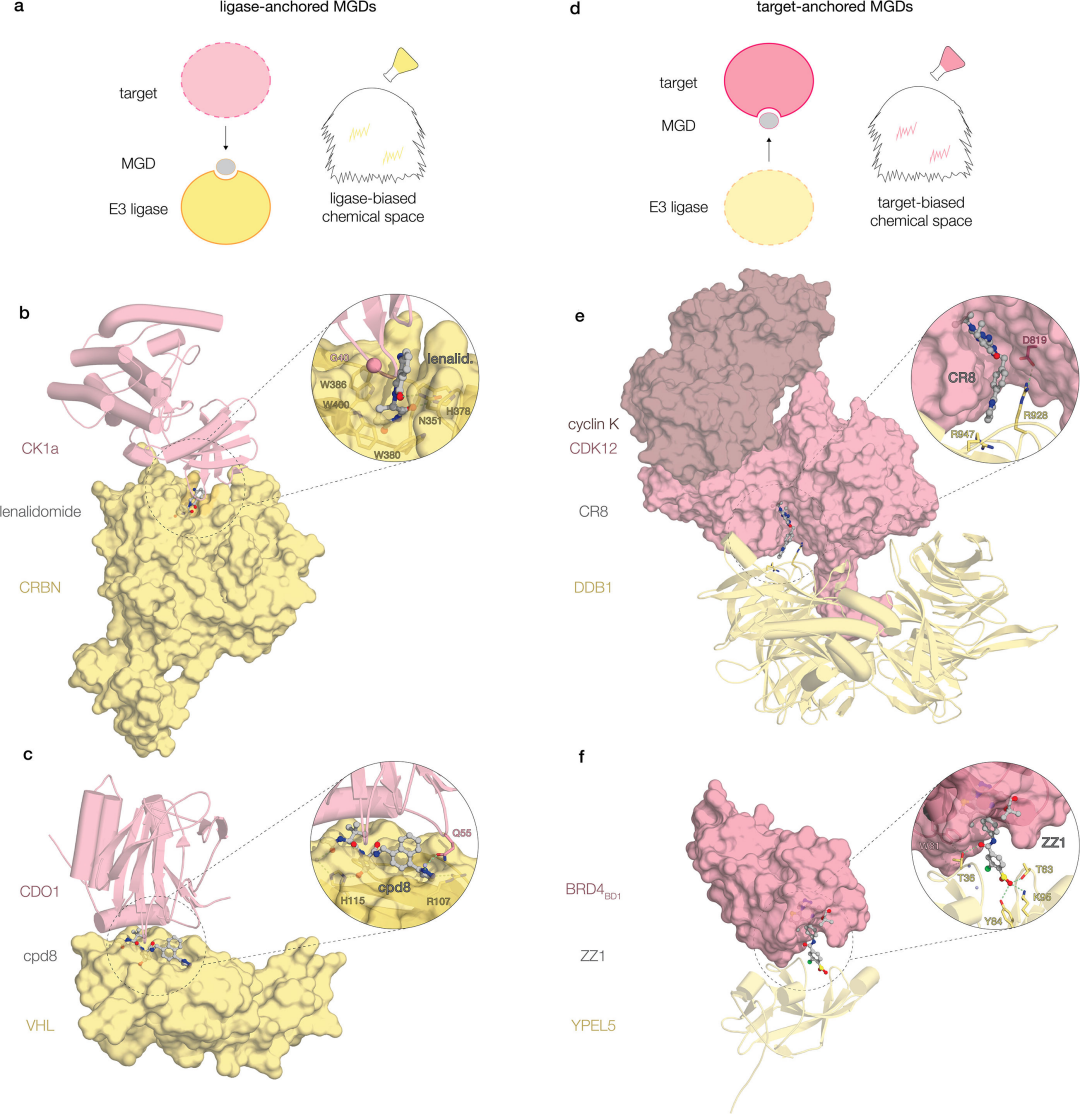
Ligase-anchored versus target-anchored molecular glue degraders. A recurring geometry in MGDs involves a ligandable pocket on one partner positioned opposite a shallow surface on the other, bridged by a small molecule. This arrangement allows many MGDs to be viewed as modular, with the primary pocket contributed either by the ligase or the target. The schematic panels (**a, d**) illustrate how the chemical search space can be biased toward such anchoring in MGD campaigns. (**a–c**) Ligase-anchored examples: (**b**) CRBN–lenalidomide–CK1α [19], (**c**) VHL–cpd8–CDO1 [34]. (**d–f**) Target-anchored examples: (**e**) DDB1–CR8–CDK12–cyclin K^33^, (**f**) YPEL5–ZZ1–Brd4_BD1_.^35^ Anchoring proteins are shown as semi-transparent surfaces, recruited partners as cartoons, and ligands in ball-and-stick representation. Ligase components are depicted in yellow and targets in pink.

The following sections explore how to tilt these odds: directed strategies that deliberately bias chemical matter – either from the ligase side or the target side – toward productive stabilization (Figure 1).

### Ligase-first strategies

The clearest example of a ligase-centric approach is CRBN, where thalidomide derivatives show how a single ligase binder can be elaborated to redirect substrate scope (Figure 1a, b). By creating a composite recognition surface, these compounds recruit targets that the ligand itself has no intrinsic affinity for [17,19]. What initially appeared narrow – the recruitment of IKZF1/3 and casein kinase 1 alpha (CK1α) via a β-hairpin G-loop – has since expanded dramatically [20,36–38]. Successive derivatives, from lenalidomide and pomalidomide to numerous next-generation analogs, have progressively broadened the neosubstrate landscape, underscoring CRBN’s capacity for reprogramming.

The determinants of CRBN recruitment have been mapped in ever finer detail. Early work delineated the zinc-finger degrome around a permissive G-loop glycine, and Słabicki and colleagues extended this using reporter screens with comprehensive zinc-finger libraries and analog panels, showing how flanking residues and subtle scaffold edits tune degradation outcomes [20,39]. Leveraging geometric deep learning, Petzold et al. [38] charted CRBN neosubstrates at the proteome scale, uncovering novel degron topologies – including helical motifs – and identifying VAV1 (Vav guanine nucleotide exchange factor 1), a substrate entirely lacking a G-loop, overturning the notion that recognition is restricted to a single motif class. In parallel, Annunziato et al. [40] showed that GTPase-activating protein-binding protein 2 (G3BP2) degradation can bypass degrons altogether by exploiting a distinct hotspot on the CRBN LON domain, together redefining CRBN as a ligase scaffold capable of supporting far more diverse recognition than previously appreciated. Beyond degron topology, conformational dynamics also shape outcomes: some substrates stabilize partially open CRBN conformations (e.g. NIMA-related kinase 7 (NEK7)), while next-generation analogs such as mezigdomide bias sensor-loop equilibria, suggesting that CULT–LON domain conformations can further modulate substrate outcomes [38,41].

Importantly, CRBN recruitment does not guarantee degradation. Many proteins are brought to CRBN but remain below the threshold for productive ubiquitination. Medicinal chemistry can shift this balance: structure-guided design and even single-atom edits have converted such recruited-but-stable proteins into bona fide substrates. IKZF2 (Helios), WIZ (widely interspaced zinc finger protein), and NEK7 exemplify this principle, and their progression into clinical programs shows how chemical elaboration of a ligase-binding pharmacophore can unlock selective degradation of otherwise resistant targets [42,43]. Together, these insights suggest that neosubstrate scope may be constrained as much by available chemotypes as by intrinsic biology. They establish CRBN as the paradigmatic case of ligase-directed enrichment: a single anchoring binder, elaborated at solvent-exposed positions, can stabilize diverse geometries and enable neomorphic recruitment.

For years, CRBN seemed unique in this malleability – until recent studies revealed that the logic can extend to other ligases. Von Hippel-Lindau (VHL), long considered primarily a PROTAC workhorse, has now been shown to support glue activity (Figure 1c). Novartis demonstrated that canonical VHL ligands such as VH032, originally designed to mimic the hydroxyproline degron of hypoxia-inducible factor 1α (HIF1α), could be elaborated into high-affinity glues that recruit the metabolic enzyme cysteine dioxygenase type 1 (CDO1), with protein-array screening and structural analyses confirming ternary complex formation [34]. Independently, Amgen constructed a VHL-focused library and, using an ultra-high-throughput Picowell RNA-seq assay, discovered dGEM3 (degrader of GEMIN3), a glue that selectively degrades the helicase GEMIN3 (Gem-associated protein 3). Biophysical and cellular studies showed that dGEM3 binds the canonical VHL pocket, while solvent-exposed substituents engage GEMIN3’s ATP-binding lobe to nucleate ternary assembly [44]. VHL thus parallels CRBN in that subtle solvent-facing modifications can redirect ligase engagement, hinting at broader reprogrammability that further chemical elaboration – and future studies – are likely to reveal.

Hence, high-quality ligase binders may provide privileged starting points and can evolve into glue platforms: with modest elaboration, they could imbue ligase surfaces with new recognition features, biasing the chemical ‘haystack’ toward productive glue discovery. As additional binders emerge – via degron mimicry (VHL, Kelch-like homology domain-containing protein 2 (KLHDC2), suppressor of cytokine signaling 2 (SOCS2)) [45–48], high-throughput discovery (DCAF1) [49], DEL screens (tripartite motif-containing protein 21 (TRIM21)) [32], or covalent approaches (RING finger protein 4 (RNF4), RNF114, feminization protein homolog B (FEM1B), DCAF16, Kelch-like ECH-associated protein 1 (KEAP1)) [50–54] – the same logic can be extended.

A broader question is which of the ~600 human ligases may prove similarly tractable once binders are available. Several recurring features appear to predispose certain families to ‘hijackability’. Geometry is a major factor: modular CRL4 ligases with a swiveling Cullin-4-DNA damage-binding protein 1 (CUL4–DDB1) arm provide a large ubiquitination zone that tolerates imperfect alignments – an architecture that pathogens have repeatedly exploited, with several viruses hijacking CRL4s to redirect degradation [55–57]. Chemistry can also create footholds: DCAF16, DCAF11, and F-box protein 22 (FBXO22) present clustered cysteines that appear to enable covalent capture, making them frequent hitters in covalent MGD campaigns [58]. It is conceivable that such reactivity might be particularly effective in ligases whose biology is already attuned to cellular redox stress, though direct evidence remains limited. Finally, inherent promiscuity may provide an advantage in some cases: ligases evolved for protein quality control – such as the TPD-validated TRIM21 or RNF126 – naturally sample broad substrate repertoires, raising the possibility that small molecules could hijack their surveillance [32,59–61]. Taken together, these features – geometry, chemistry, and promiscuity – may help rationalize why certain ligases repeatedly surface in glue campaigns and suggest patterns that could merit further exploration.

### Target-first strategies

Although ligases have provided the clearest entry point so far, in most areas of drug discovery efforts begin with the target: a defined vulnerability for which compounds are sought. In MGDs, by contrast, the earliest targets were not prospectively selected, but recognized only in retrospect.

The case of CR8 offered a different perspective, showing how target anchoring can serve as a starting point for MGD discovery (Figure 1d, e). This kinase inhibitor binds cyclin-dependent kinase 12 (CDK12) and reshapes its surface to recruit DDB1, triggering selective degradation of the co-activator cyclin K. The resulting ternary complex represented a notable departure from precedent: in the absence of a canonical DCAF, the kinase itself acted as a drug-induced substrate receptor, and – as later revealed through extensive structural work – the same interaction could be supported by a wide range of chemotypes capable of bridging the interface [28,30,31,33,62–65]. A key design insight emerged from comparison with its parent compound, roscovitine, which remains a conventional inhibitor [33,66]. The two scaffolds differ solely in a solvent-exposed pyridine substituent that, in the ternary structure, protrudes toward DDB1. That single outward-facing ring appeared sufficient to switch the degradation activity on, showing how surface-oriented elaboration can endow inhibitors with gain-of-function activity. What first looked like a curious inversion of glue logic – a compound with affinity for its target recruiting the ligase *de novo*, rather than the reverse – emerged instead as a generalizable strategy. A similar toggle was observed with B-cell lymphoma 6 (BCL6) ligands: BI-3812 acts as a conventional BTB (Broad-Complex, Tramtrack, and Bric-à-brac)-domain inhibitor, whereas its close analog BI-3802, differing only by a solvent-exposed substituent, induces BCL6 polymerization and degradation via its endogenous ligase seven in absentia homolog 1 (SIAH1) [67,68]. These early insights spurred more deliberate campaigns.

One example of prospective MGD discovery came from the compound ZZ1 (Figure 1f) [35]. Derived from JQ1 through installation of a sulfonyl fluoride – one of only a few dozen analogs modified on solvent-exposed exit vectors in this study – ZZ1 was found to induce bromodomain-containing protein 4 (BRD4) degradation through the glucose-induced degradation/C-terminal to LisH (GID/CTLH) ligase, a non-CRL complex not previously hijacked for targeted degradation. Structural analyses revealed a glue-induced interaction of BRD4 with the Yippee-like protein 5 (YPEL5) subunit, where the metabolically converted sulfinic acid moiety projected toward the ligase. This provided compelling evidence that solvent-facing elaboration of known binders can yield MGDs, while also serving as a strategy to uncover hijackable ligases outside the canonical CRL family.

Covalent handles represent a related extension of this logic: electrophilic tags such as acrylamides, fumarates, or aldehydes could be grafted onto diverse inhibitors to channel degradation via recurring ligases [58,60,63,69–71]. Their mechanisms, however, appear heterogeneous – ranging from PROTAC-like induced proximity to template-assisted covalent gluing – and further structural work will be important to better define the circumstances under which covalency may substitute for, or complement, co-operative recognition.

Taken together, these examples suggest that target-first approaches provide a complementary starting point: subtle solvent-facing elaborations – whether noncovalent or covalent – can convert conventional binders into degraders, though such strategies work best when proteins already harbor some latent structural compatibility.

## Predisposed interfaces

This raises the deeper question: which protein–protein interfaces are naturally most suited to glue action, and how can we recognize them in advance? This review approaches the question in three steps. First, mimicry provides a biological lens, showing how glues can echo cues already used in nature. Second, biophysics offers the constraints, helping to explain when weak compatibilities can be stabilized. Third, from these perspectives emerge signposts – practical heuristics that can help translate concept into discovery.

### Mimicry

A recurring theme across MGDs is that their activity rarely depends on inventing recognition surfaces *de novo*. Instead, most cases can be rationalized as forms of mimicry, whereby small molecules reproduce signals that biology already uses to regulate protein fate.

**Figure 2:**
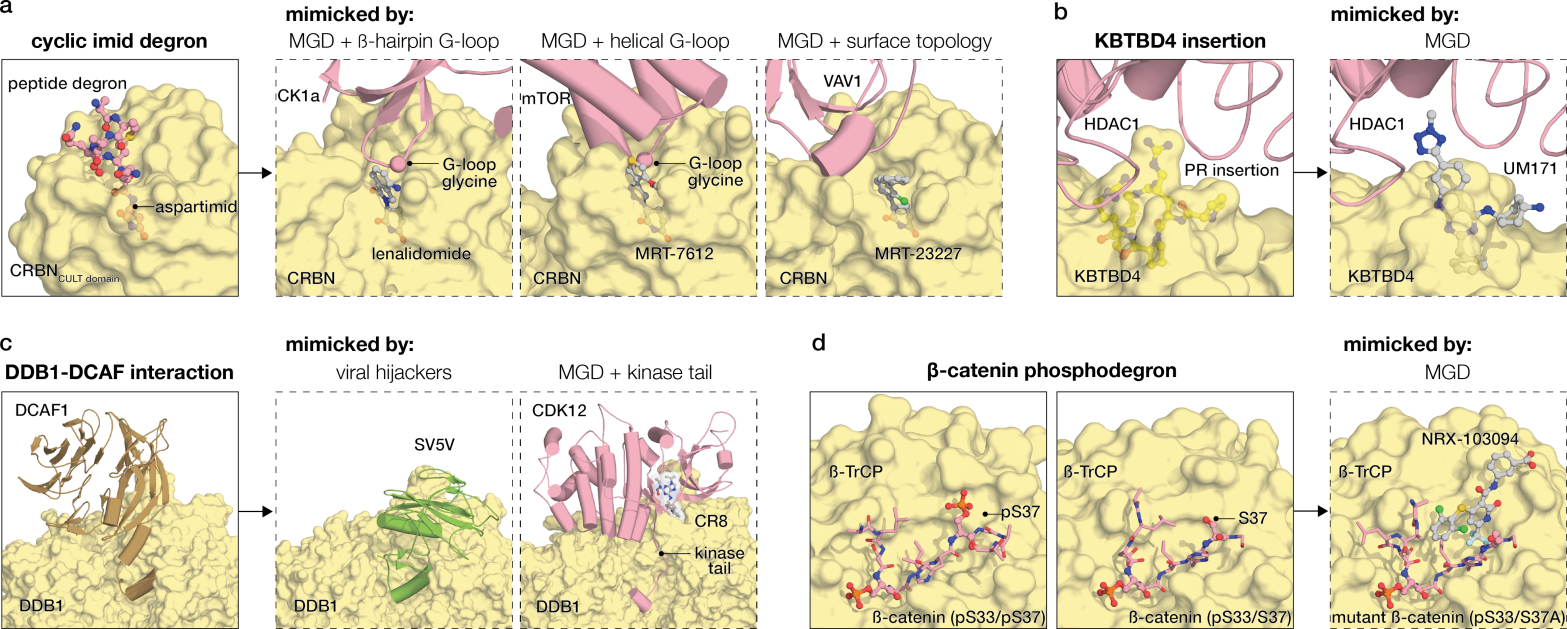
Mimicry as a unifying principle in molecular glue degradation. MGDs can act by mimicking native molecular features that drive ligase–target recognition. (**a**) Thalidomide analogs: native post-translational modification degrons compared with recruited substrates displaying analogous structural motifs – CK1α β-hairpin G-loop [19], mTOR helical G-loop [38] – and VAV1, bearing no structural similarity except for surface topology [38]. CRBN is displayed in yellow and targets in pink. (**b**) Cancer mutation mimicry: neomorphic KBTBD4 mutant interfaces compared with UM171 engagement of the same KBTBD4 surface [72]. KBTBD4 is displayed in yellow, with the PR insertion loop shown in ball-and-stick representation. HDAC1 is shown in pink. (**c**) DCAF mimicry: native DCAF1-DDB1 recognition [73] compared with viral hijacking of DDB1 [74] and CR8-mediated conversion of CDK12 into a drug-induced substrate receptor [33]. DDB1 is shown in yellow, DCAF1 in brown, SV5V in green, and CDK12 in pink. (**d**) Repair of a broken degron: a mutation-impaired phosphodegron can be repaired with a small molecule that restores recognition. β-TrCP is shown in yellow and β-catenin in pink, with phosphoserines and the compound shown in ball-and-stick representation. Abbreviations: PR, proline arginine; HDAC1, histone deacetylase 1.

Thalidomide analogs provide the clearest demonstration (Figure 2a). For many years, the evolutionary rationale for the thalidomide-binding cavity of CRBN was obscure, prompting searches for endogenous CRBN binders or unknown facets of CRBN biology. Recent work from the Woo and Hartmann laboratories resolved this puzzle, showing that the tri-tryptophan pocket of CRBN recognizes endogenous cyclic imide degrons formed at protein termini [75–78]. IMiDs exploit this natural recognition module: the glutarimide anchors in the cavity, while the phthalimide projects outward toward neosubstrates. In this way, they act as chemical stand-ins for a native degron, thus redirecting CRBN specificity.

UM171 offers a complementary lesson (Figure 2b). Identified in a phenotypic screen for hematopoietic stem-cell expansion, UM171 was later shown to converge mechanistically with pathogenic Kelch repeat and BTB domain-containing protein 4 (KBTBD4) insertions in medulloblastoma [29,72,79,80]. Both the compound and the mutations create a neo-interface that promotes CoREST (corepressor for element-1 silencing transcription factor) degradation via KBTBD4 [72,80]. Structural analysis revealed that the same loop on KBTBD4 that is altered by mutation can also be engaged by UM171, yielding an identical gain-of-function outcome [72]. This parallel underscores that mutations in ligases serve as natural experiments in reprogrammability: both a compound and a genetic lesion can endow a ligase with new recognition capacity.

Other glues show less intuitive forms of mimicry. CR8, for instance, when bound to CDK12, remodels the kinase into a pseudo-substrate receptor, exposing an adaptor-like helix–loop–helix motif that engages DDB1 and mimics the geometry of a canonical DCAF [33,57]. This is the same pliability that viral proteins exploit: the hepatitis B virus X protein (HBx) and the V protein of Simian Virus 5 (SV5V) both co-opt DDB1 via helix–loop–helix motifs, closely mirroring the pseudo-DCAF geometry of glue-bound CDK12 (Figure 2c) [57,74]. Together, these cases underscore that both small molecules and pathogens exploit the inherent reprogrammability of ligase surfaces – modular architectures and permissive geometries that make them naturally suited for engaging neosubstrates.

Beyond degrons and post-translational modifications (PTMs), some glues mimic the interaction modules that substrates normally use with native partners. G3BP2, for example, recruits proteins such as USP10 and Caprin1 via its NTF2L (nuclear transport factor 2-like) domain, where short FGDF/FxFG motifs dock into a conserved hotspot on the dimer [81,82]. In the glue-induced CRBN–G3BP2 ternary complex, that same hotspot is engaged – not through CRBN’s canonical degron pocket, but via an alternative surface stabilized by the small molecule MRT-5702 [40]. Rather than inventing a new interface, the glue co-opts recognition logic already encoded in G3BP2 biology, showing that mimicry can extend from degron grammars to whole protein-protein interaction (PPI) modules.

From this perspective, mimicry is not just an explanatory frame but a design heuristic. Endogenous degrons, disease mutations, viral hijackers, and topologies of other PPIs all reveal geometries naturally suited to reprogramming. Small molecules can act as chemical surrogates for these cues, stabilizing weak compatibilities into productive recognition events.

### Rules of engagement

If mimicry helps explain *why* molecular glues work, interface properties clarify *how* they succeed. Evidence suggests that glues most often stabilize weak, pre-existing compatibilities [83]. In several cases – including CRBN–CK1α, DDB1–CDK12, and DCAF15–RBM39 – the ligase and substrate interact weakly on their own, typically at micromolar affinity, with the small molecule shifting this into a tighter, nanomolar complex. In others, such as VHL–CDO1 or CRBN–G3BP2, no measurable binary affinity is observed, yet ternary assembly emerges once a ligand contributes sufficient co-operativity. Parallel evidence from Garcia-Seisdedos et al. [84] showed that random surface mutations often generate weak, new PPIs in the µM–mM range, suggesting that protein surfaces are populated with ‘quasi-interfaces’ that, though usually silent, can be stabilized.

Whether such weak compatibilities can be made productive depends on their energetic architecture. Native PPIs concentrate binding free energy in a few hotspot residues, buffered by an O-ring of surrounding contacts that shield them from solvent and modulate robustness [85–87]. Classic alanine scanning studies (human growth hormone–human growth hormone receptor, Ras–Raf, PDZ–peptide, antibody–antigen) established this logic: mutating a hotspot sharply weakens affinity, whereas peripheral substitutions are more often tolerated [86,88–90]. By contrast, glue-induced complexes usually lack such buffering. Structural and mutational studies show that stability can hinge on just a few ligand-defined contacts, with single substitutions sufficient to abolish recruitment – a brittleness confirmed by deep mutational profiling [91]. This helps explain why glue mechanisms often display steep structure-activity relationship (SAR) and strong specificity, and why productive degradation can depend sensitively on cellular context.

Recent studies also suggest that ligands can act as artificial O-rings for protein–protein interactions, stabilizing fragile polar bonds between ligase and substrate by shielding them from solvent [92]. In CRBN–lenalidomide–CK1α, the compound reinforces pre-existing hydrogen bonds; CR8 shields a CDK12–DDB1 salt bridge; and the CDO1 degrader forms a hydrophobic rim that buttresses polar interactions [34,62,92]. These cases highlight how small molecules can enhance otherwise marginal PPI contacts directly, tipping them over the energetic threshold for stable recognition [93].

Viewed across systems, one recurring geometry stands out. Glueable sites often feature a ligandable pocket on one protein positioned opposite a relatively featureless surface on the other. The pocket – such as CRBN’s tri-tryptophan cavity or the ATP-binding cleft of CDK12 – serves as an anchor for the small molecule, while the opposing surface alone is too weak to support binding (Figure 1). This arrangement has been proposed as a practical design heuristic, echoing the modular view introduced earlier [40,62]. While exceptions exist – true interface stabilizers such as NRX-103094 or HQ461, which act without appreciable affinity for either protein – these remain rare compared with the dominant pocket-anchored logic [30,62,94].

Within this arrangement, ternary complexes can be grouped into two archetypes [91]. Motif–domain assemblies (e.g. CRBN–neosubstrates, DCAF15–RBM39) bury relatively small surfaces (~500–900 Å²) and depend heavily on ligand contribution, which helps explain their steep SAR and, in the case of CRBN, the relative ease of substrate redirection. Domain–domain assemblies (e.g. CDK12–DDB1, VHL–CDO1) bury much larger surfaces, with interactions dominated by protein–protein contacts, leading to flatter SAR and narrower substrate scope. These comparisons suggest that lessons from thalidomide analogs do not necessarily generalize across all glueable systems: each interface follows its own co-operative logic, underscoring the need for systematic structural exploration.

A distinct variation comes from covalent ‘chemical degrons’. Electrophilic handles can covalently tether a ligase to its partner, bypassing some of the co-operativity required for noncovalent glues. This may explain why such moieties can sometimes be transplanted across scaffolds, in certain cases functioning like minimal PROTAC-style warheads [51,60]. At the same time, they span a mechanistic spectrum: some rely mainly on enforced geometry, while others (e.g. GNE011) act through template-assisted mechanisms in which covalent reactivity is primed by pre-existing compatibilities [70]. Defining, at the structural level, the contexts in which covalency substitutes for, or augments, co-operative recognition may provide important insight.

Across diverse examples, data support a threshold model: ternary assembly becomes efficient only once the composite interface clears a minimal energetic barrier. This framing in turn helps connect recurring features of glues: systems with steep SAR or plastic specificity probably sit close to this line, where small changes can tip the balance, whereas flatter SAR or fixed specificity reflect assemblies stabilized well above it. From a design standpoint, the question becomes: where in the proteome should we search for interfaces poised near this boundary?

### Glueable blueprints

The search space – tens of thousands of proteins and ~600 ligases – may seem overwhelmingly vast. Yet, glueable pairs do recur, hinting that latent compatibilities may be more common than initially assumed, and that small molecules can, under certain conditions, exploit them.

Known protein–protein interactions provide pre-existing affinity, while endogenous ligase–substrate pairs are already wired for ubiquitination and thus offer especially clear entry points. Many of these are tuned by degron ‘toggles’, whereby recognition is switched on or off by minimal cues such as hydroxylation (VHL), phosphorylation (β-TrCP (β-transducin repeat-containing protein) or FBXW7 (F-box and WD repeat domain-containing 7)), or endogenous small molecules [95–98]. Auxin, for instance, bridges TIR1 (transport inhibitor response 1) and Aux/IAA (auxin/indole-3-acetic acid) to stabilize an otherwise weak encounter, while nuclear hormone receptors expose degrons in response to ligand binding – a principle exploited therapeutically by selective estrogen receptor degraders [99–101]. The broader logic is that biology already encodes conditional interfaces, and in some cases, small molecules may substitute for these natural cues to stabilize otherwise transient recognition. Systematically charting such weak or short-lived ligase–substrate contacts – often invisible in standard interactome maps – could provide a practical blueprint for the discovery of MGDs.

Genetics offers a second kind of signpost. Mutations act as natural perturbations, exposing interfaces on the edge of stability. The β-catenin phosphodegron is a canonical case: oncogenic mutations at S37 disrupt phosphorylation and abolish SCF (Skp1-Cullin-1-F-box)^β-TrCP^ recognition, leaving the degron geometrically intact but chemically incomplete [102]. Simonetta et al. [94] showed that small molecules can ‘patch’ this hypomorph by mimicking the missing phosphoserine, restoring degradation (Figure 2d). A similar principle applies to SMAD4 (Mothers against decapentaplegic homolog 4), where recurrent R361H mutations weaken SMAD4–SMAD3 dimerization; small molecules can act as prosthetics for the lost side-chain chemistry [103]. Protective alleles illustrate the inverse, as with the CARD9_Δ11_ (caspase recruitment domain-containing protein 9, exon 11 deletion) splice variant that removes a TRIM62 binding site – logic that small molecules can also mimic allosterically [104]. Some lesions go further, creating neomorphic interfaces. UM171 phenocopies pathogenic KBTBD4 insertions that reprogram CoREST recruitment (Figure 2b) [72,80]. Other recurrent cancer mutations highlight similar opportunities: BRAF_V600E_ generates a degron-like patch that engages KEAP1, while mutations in the SPOP (speckle-type POZ protein) MATH (meprin and TRAF homology) domain rewire its substrate specificity [105–108]. Together, these examples suggest that even single amino acid changes can uncover latent compatibilities – of the sort that small molecules may be able to render productive. Mutational landscapes may therefore provide a rich, underexplored resource for glue discovery.

A final guide comes from structural ‘glueprints’. Every ternary structure solved to date does more than rationalize a single mechanism – it encodes a grammar of recognition, showing how ligand, ligase, and substrate can be stitched together into a productive interface. Some of these grammars already show signs of portability: CRBN’s G-loop logic appears across multiple substrates (Figure 2a), while CDK12’s pseudo-DCAF geometry echoes strategies used by viral proteins to hijack DDB1 (Figure 2c). Monte Rosa’s identification of VAV1 as a CRBN neosubstrate illustrates this portability: although VAV1 bears no resemblance to known substrates, it mimicks the topology of G1 to S phase transition protein 1 (GSPT1)’s G-loop [38]. Going a step further, CRBN-mediated degradation of G3BP2 exploits a hotspot active in G3BP2’s natural PPI network, hinting that interactome context may help highlight glueable surfaces [40]. In both cases, glues do not create entirely new surfaces but instead co-opt recognition logics already embedded in the proteome. As structural catalogs expand – and with computational tools such as geometric deep learning beginning to mine them – emerging principles may increasingly guide systematic recognition of glueable interfaces.

## Outlook

Molecular glues highlight a simple but consequential idea: small molecules can act as ‘chemical PTMs’ or ‘chemical mutations’, reprogramming recognition rather than only blocking activity [109]. The features they impose on protein surfaces – an added hydrogen-bond donor, a new hydrophobic patch, a shielded hotspot – are similar to what surface mutations or phosphorylation can achieve (Figure 2). Biology relies on such minimal perturbations to tune interactions, creating neoepitopes that redirect recognition networks. Seen in this light, glues may be less outliers than echoes of strategies that nature already employs – a framing that can inform systematic discovery.

From the examples reviewed here, several heuristics emerge. First, glueable sites are often defined by a common geometry: a ligandable pocket on one partner positioned opposite a shallow patch on the other, where a small molecule can anchor and stabilize marginal contacts. Even weak micromolar affinities can be nudged into stability by such co-operativity, and fragile polar bonds can be reinforced through hydrophobic shielding. Second, biology leaves additional clues: mimicry of PTMs, mutations, or viral hijacks reveals recognition logics that glues can borrow, while recurrent lesions point to ligases unusually pliable to redirection. Finally, structural precedent offers another guide: every solved ternary complex leaves behind a ‘glueprint’, a transferable grammar of recognition that can, in principle, be redeployed elsewhere. Notably, these notions apply to molecular glues more broadly. Recent examples such as 14-3-3 interaction stabilizers, SLFN12–PDE3A (Schlafen family member 12-phosphodiesterase 3A) glues, and KRAS–cyclophilin recruiters show how small molecules can redirect protein–protein interactomes with outcomes extending beyond protein degradation, underscoring the wider promise of using chemistry to rewire recognition networks [110–112].

Taken together, these directions mark a shift in emphasis: from searching the haystack for rare positives to mapping and leveraging the latent compatibilities already embedded in protein recognition. With expanding chemical space and deeper biological insight, glue discovery may increasingly move from serendipity to design – not by inventing recognition from scratch, but by leveraging cues already inscribed in protein networks.

**Summary Points**

- Molecular glue degraders (MGDs) harness the ubiquitin–proteasome system to eliminate proteins that may be otherwise difficult to target with conventional inhibitors.
- Structural studies reveal recurring recognition principles – anchored pockets, degron mimicry, and transferable ‘glueprints’ – that help explain how glues work.
- Discovery can be guided by both chemical entry points (ligase or target binders) and biological cues (native degrons, genetic lesions, viral hijacks).
- MGDs may increasingly be understood not as rare anomalies but as echoes of recognition strategies already embedded in the ubiquitin system.
- Future progress may come from mapping latent ligase–substrate compatibilities, expanding chemical space, and deepening structural insight into diverse glue-mediated assemblies.

## Open Access

This article has been published open access under our Subscribe to Open programme, made possible through the support of our subscribing institutions, learn more here: https://portlandpress.com/pages/open_access_options_and_prices#conditional

## Abbreviations

MGDs: molecular glue degraders
PROTAC: proteolysis-targeting chimeras
TPD: targeted protein degradation
UPS: ubiquitin–proteasome system
